# Identification of Key Biomarkers and Pathways in Small-Cell Lung Cancer Using Biological Analysis

**DOI:** 10.1155/2021/5953386

**Published:** 2021-10-19

**Authors:** Huanqing Liu, Tingting Li, Xunda Ye, Jun Lyu

**Affiliations:** ^1^Department of Clinical Research, The First Affiliated Hospital of Jinan University, Guangzhou, Guangdong, China; ^2^Department of Pharmacy, Xi'an Chest Hospital, Xi'an, Shaanxi, China; ^3^Clinical Medicine Research Institute, The First Affiliated Hospital of Jinan University, Guangzhou, Guangdong, China

## Abstract

**Background:**

Small-cell lung cancer (SCLC) is a major cause of carcinoma-related deaths worldwide. The aim of this study was to identify the key biomarkers and pathways in SCLC using biological analysis.

**Methods:**

Key genes involved in the development of SCLC were identified by downloading three datasets from the Gene Expression Omnibus (GEO) database. Differentially expressed genes (DEGs) were screened using the GEO2R online analyzer; for the functional annotation and pathway enrichment analysis of genes, Funrich software was used. Construction of protein-to-protein interaction (PPI) networks was accomplished using the Search Tool for the Retrieval of Interacting Genes (STRING), and network visualization and module identification were performed using Cytoscape.

**Results:**

A total of 268 DEGs were ultimately obtained. The enriched functions and pathways of the upregulated DEGs included cell cycle, mitotic, and DNA replication, and the downregulated DEGs were enriched in epithelial-to-mesenchymal transition, serotonin degradation, and noradrenaline. Analysis of significant modules demonstrated that the upregulated genes are primarily concentrated in functions related to cell cycle and DNA replication. Kaplan-Meier analysis of hub genes revealed that they may promote the carcinogenesis and progression of SCLC. The result of ONCOMINE demonstrated that these 10 hub genes were significantly overexpressed in SCLC compared with normal samples.

**Conclusion:**

Identification of the molecular functions and signaling pathways of participating DEGs can deepen the current understanding of the molecular mechanisms of SCLC. The knowledge gained from this work may contribute to the development of treatment options and improve the prognosis of SCLC in the future.

## 1. Background

Lung cancer is a major cause of carcinoma-related deaths worldwide, and approximately 2.21 million new cases of this disease are estimated in 2020 according to the latest WHO data on lung cancer. Gene mutations and cell environment changes may affect the formation, growth, and metastasis of tumors [1]. Small-cell lung cancer (SCLC) is the main histological form of pulmonary carcinoma. Conventional treatment methods include chemotherapy, radiotherapy, and surgery. Chemotherapy is the most important treatment method for SCLC, but issues such as high drug resistance and recurrence rates limit its effectiveness. Because most patients are usually at advanced stages of the disease at the time of diagnosis, SCLC is often characterized by low survival rates and poor quality of life. In fact, SCLC has a 5-year survival rate of <6% and high mortality; moreover, it is highly invasive and prone to early hematogenesis and lymphatic metastasis [2]. To date, no molecular targeted drugs have yet been shown to significantly prolong patient survival [3]. More importantly, the molecular mechanisms underlying the occurrence, development, invasion, and metastasis of SCLC remain poorly understood. Thus, finding methods to obtain a prognosis of SCLC and identify potential biomarkers for targeted therapy is of great importance to improve the clinical efficacy of lung cancer.

Advances in gene expression profile chip technology over the last few decades have established a strong foundation for the overall exploration of differentially expressed genes (DEGs) in lung cancer and their biological functions. Studies have shown that the expression profiles of cancerous tissues differ from those of neighboring noncancerous tissues [4]. Given theoretical facts on cancer and medical database as well as data mining technology in big data era, we rationally speculated that DEGs may influence the occurrence and development of many diseases, including malignancies. Although some RNA-sequencing (RNA-seq) are insensitive to ribonuclease because of their unique structure, different genes can exist in tissues and serum and possibly even function as biomarkers for cancer. Microarray and bioinformatics technologies show broad applications in disease research, especially for identifying DEGs, mRNA, and miRNA, as well as their elucidation of molecular mechanisms [5–7]. Udhaya et al. used bioinformatics methods to identify four DEGs and associated pathways in systemic lupus erythematosus [8]. Many non-small-cell lung cancer (NSCLC) gene expression profile studies have been conducted using microarray technology, and numbers of NSCLC-related DEGs have been identified [9]. Mao et al. used bioinformatics methods to compare the differential expressions of mRNA and microRNA between SCLC tissues and normal lung tissues to explore the pathogenesis and potential molecular markers of SCLC [10]. Due to the complexity of the biological characteristics of SCLC, the key biomarkers and specific targets for prognosis of SCLC remain unclear. Therefore, it is necessary to explore more genetic information and screen out potential or promising biomarkers for prognosis of SCLC. Chen et al. identified 8 DEGs served as new biomarkers for prognosis of SCLC [11]. However, due to the lack of SCLC-related gene chip data, we need to integrate more chip database for research and analysis of this disease. In the current study, three gene expression profiles, i.e., GSE30219, GSE99316, and GSE149507, were downloaded from public databases to identify DEGs in SCLC and their related pathways. Moreover, Kaplan-Meier analysis was used to explore the relationship between prognosis and hub gene expression level, and the Oncomine database was used to explore hub genes' expressions between SCLC tissues and normal tissues. The obtained data indicate that the identified DEGs may be used as key biomarkers of SCLC. The related pathways also offer insights into the pathogenesis of the disease.

## 2. Materials and Methods

### 2.1. Microarray Data

Gene Expression Omnibus (GEO; http://www.ncbi.nlm.nih.gov/geo/) was adopted to perform gene chip screening. The target chip access criteria are as follows: (1) clinical SCLC specimens of the patients were excluded from cell lines and animal experiments. (2) The selected chip should contain SCLC and normal tissue samples. (3) Only mRNA chips that have been standardized were employed. According to the screening criteria, several datasets were screened out. We selected the datasets with large numbers of samples as the research object. Three gene profiles, i.e., GSE30219, GSE99316, and GSE149507, were then obtained from the GEO database. The GSE30219 dataset contained 21 SCLC and 14 normal tissue samples, the GSE99316 dataset consisted of 23 SCLC and 42 normal tissue samples, while the GSE149507 dataset consisted of 18 SCLC and 18 normal tissue samples.

### 2.2. Identifications of DEGs

We used the GEO2R (http://www.ncbi.nlm.nih.gov/geo/geo2r) statistical tool to recalculate and evaluate genes expressed differently in human SCLC tissues and adjacent noncancerous lung tissues.

Benjamini and Hochberg (error detection rate) and *t*-test methods were used in conjunction with GEO2R to calculate FDR and *P* values, respectively, to identify DEGs. The DEGs were confirmed by the following criteria: *P* < 0.05 and ∣log FC | >1. If logFC > 1, the gene expression is considered upregulated; if logFC < −1, the gene is considered downregulated.

### 2.3. Functional Enrichment Analysis of DEGs

Funrich (http://www.funrich.org/), a stand-alone software tool used mainly for functional enrichment and interaction network analysis of genes and proteins, can help users load a customized database against which functional enrichment analysis can be carried out. In our study, molecular function, biological process, cell composition, and biological pathway was applied to analyze the DEGs.

### 2.4. Construction of PPI Networks and Module Analysis

Search Tool for the Retrieval of Interacting Genes version 11.0 (STRING; http://string-db.org) is an online tool that could be used to identify the interactions of proteins and obtain insights into the mechanisms of certain diseases. In the present study, an overall score > 0.4 was set as the cut-off point. Cytoscape version 3.7.1 software was used to visualize the Protein–proteininteractions (PPI) networks. Molecular Complex Detection version 1.6.1 (MCODE), a Cytoscape plug-in, can confirm areas with dense connections to select a statistically significant model. Key modules within the PPI networks were identified using MCODE (MCODE score > 5, degree cut-off = 2, node score cut-off = 0.2, max depth = 100, *k*‐score = 2). Enrichment analysis of the DEGs in this module was subsequently conducted using Funrich.

### 2.5. Selection and Analysis of Hub Genes

The top 10 genes in significant modules were selected as hub genes. The cBioPortal online platform was employed to analyze the gene networks obtained, as well as the relationships among coexpressed genes. Biological Networks Gene Oncology version 3.0.3, another Cytoscape plug-in, was used to assess the performance and visualize the results of the bioprocess analysis for the hub genes. The UCSC Cancer Genomics Browser (http://genome-cancer.ucsc.edu) was also utilized to achieve the hierarchical clustering of these genes. We used Kaplan-Meier analysis to explore the relationship between prognosis and hub gene expression level and draw the corresponding survival curves. The ONCOMINE database was used to analyze the expression of the hub gene between tumor and normal tissues in clinical SCLC and the specific expression at each stage by histogram.

### 2.6. Statistical Analysis

*P* values and FDR were calculated using GEO2R's built-in *t*-test, Benjamini and Hochberg (false detection rate), and other methods to determine the DEGs of SCLC patients and controls.

## 3. Results

### 3.1. Identification of DEGs

**Figure 1:**
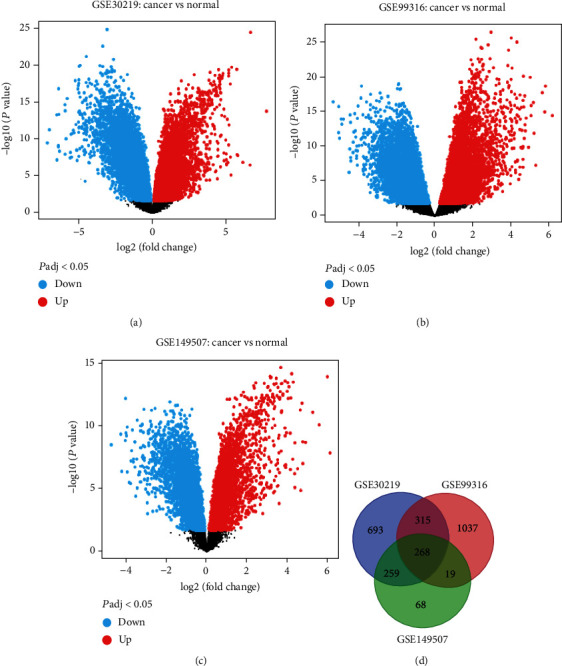
Identification of differentially expressed genes (DEGs) between SCLC and normal tissues. (a) Volcanic distribution map of DEGs in GSE30219 dataset. (b) Volcanic distribution map of DEGs in dataset GSE99316. (c) Volcanic distribution map of DEGs in the GSE149507 dataset (green indicates low expression, red indicates high expression, and black indicates no difference). (d) A Venn diagram shows the DEGs with three datasets overlapping.

According to the inclusion criteria, a total of two mRNA microarray datasets that met the requirements were screened out, namely, GSE30219, GSE99316, and GSE149507. A total of 2659 DEGs (1534 in GSE30219, 1639 in GSE99316, and 614 in GSE149507) (Table S1) were identified from the three datasets. GEO2R analysis showed that 268 DEGs were expressed in the three datasets; Venn diagram analysis of these genes is shown in Figure 1, including 192 upregulated and 76 downregulated genes in both SCLC and normal tissues (Table S2); the list of DEGs were shown in Table 1.

### 3.2. Functional Enrichment Analyses of DEGs

**Figure 2:**
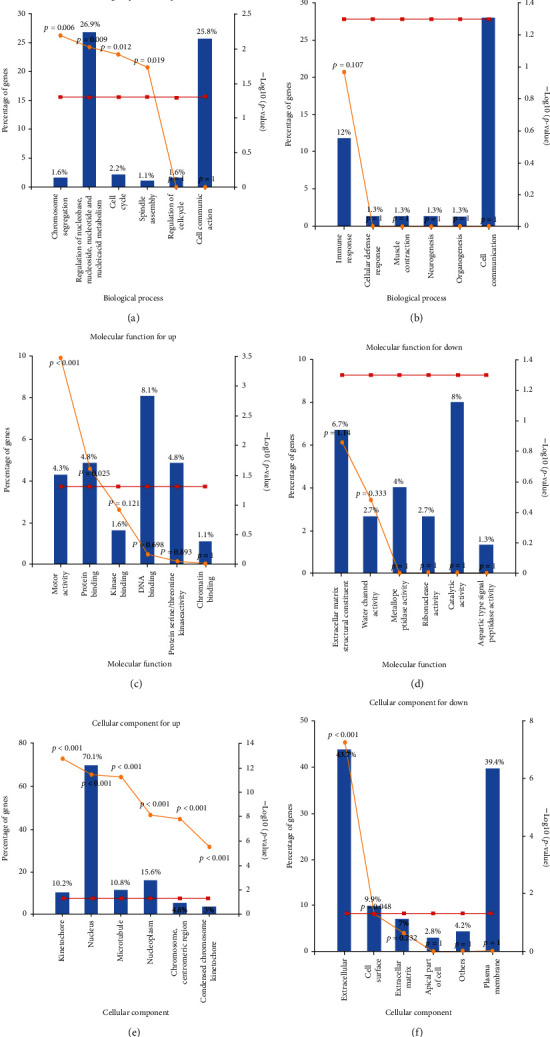
Functional annotation and path enrichment analysis. (a) BP enriched upregulated DEGs. (b) BP enriched downregulated DEGs. (c) MF enriched upregulated DEGs. (d) MF enriched downregulated DEGs. (e) CC enriched upregulated DEGs. (f) CC enriched downregulated DEGs. The *X* axis is a detailed term for functional annotation and path enrichment. The *Y* axis represents the percentage of genes, log10(*P* value). The red line is *P* = 0.05, which is the reference value of statistical analysis truncation standard. The yellow line represents log10(*P* value).

The results of GO (including three categories: biological processes (BP), cellular components (CC), and molecular functions (MF)) and biological pathway analyses of these DEGs are shown in Figure 2 (Table S3, S4). The upregulated DEGs related to BP included those for chromosome segregation, regulation of nucleobase, nucleoside, nucleotide and nucleicacid metabolism, cell cycle, and spindle assembly. Most of the upregulated DEGs obtained were related to MF, including motor activity and protein binding. The upregulated DEGs related to CC were mainly located in kinetochore, nucleus, microtubuls, nucleoplasm, chromosome, centrometric region, and condensed chromosome kinetochore.

While the analysis of BP in downregulated DEGs were most but not significantly concentrated in immune response, cell communication, and cellular defense response, most of the downregulated DEGs obtained were related to MF, including extracellular matrix structual constituent, water channel activity, and catalytic activity (there was no statistical significance). The downregulated DEGs related to CC were mainly located in extracellular and cell surface.

**Figure 3:**
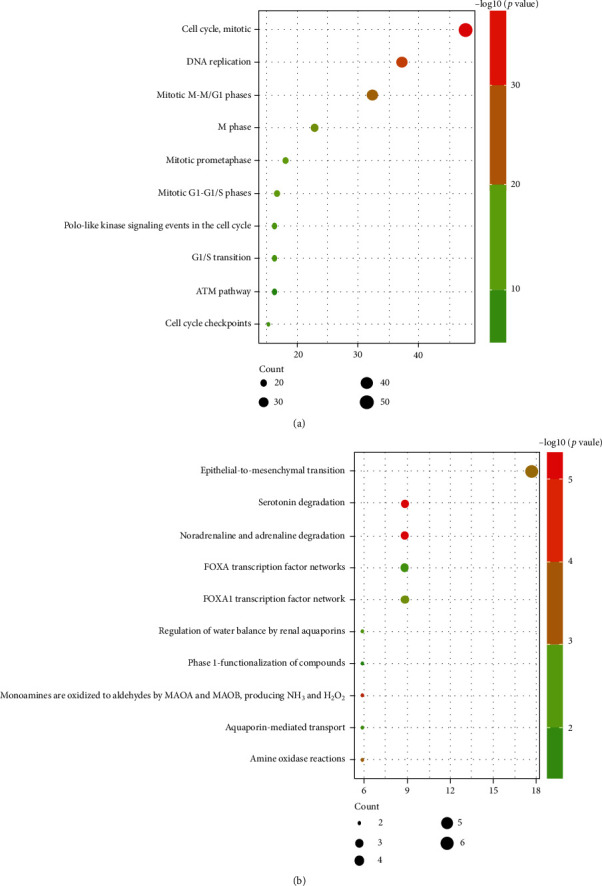
Enrichment of biological pathway analysis of DEGs. *P* value represents the color depth of nodes. The size of nodes means the quantity of genes. (a) Upregulated DEG terms. (b) Downregulated DEG terms.

Furthermore, biological pathway analysis was shown in Figure 3; the upregulated DEGs were mainly enriched in cell cycle, mitotic, DNA replication, mitotic M-M/G1 phases, M phase, mitotic prometaphase, and mitotic G1-G1/S phases. The downregulated DEGs were mainly enriched in epithelial-to-mesenchymal transition, serotonin degradation, noradrenaline and adrenaline degradation, FOXA transcription factor networks, and FOXA1 transcription factor network.

### 3.3. Construction of PPI Networks and Significant Module Analysis

**Figure 4:**
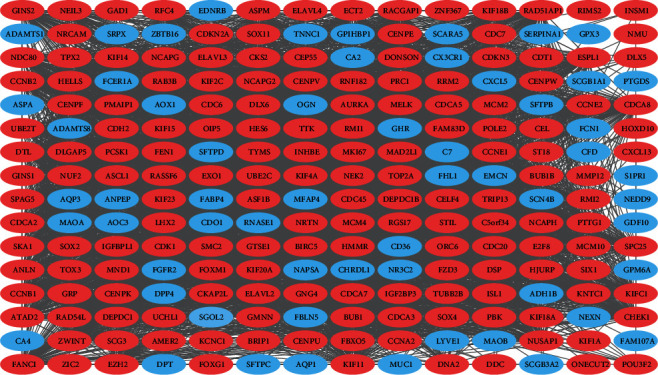
Cytoscape was utilized for constructing the PPI network of DEGs. Light red was applied for marking the upregulation genes. The downregulation genes are labeled in blue.

**Figure 5:**
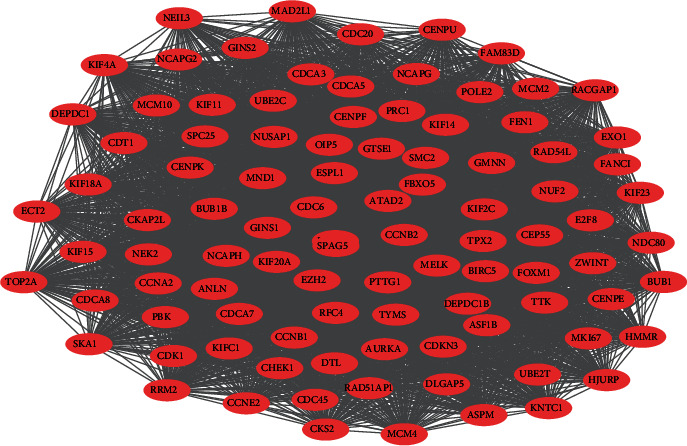
The foremost module was acquired from the PPI network.

**Figure 6:**
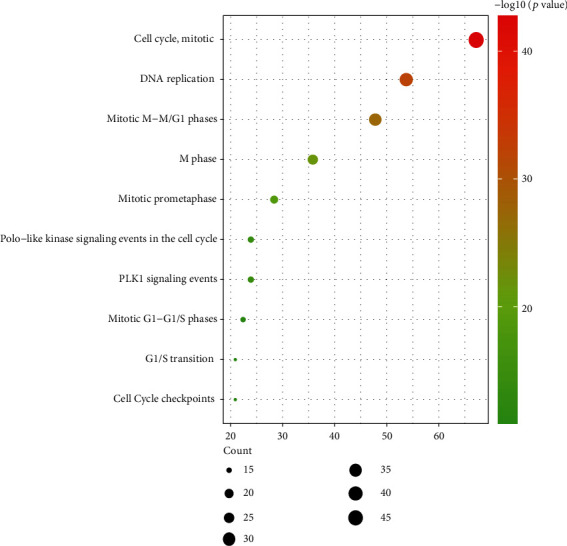
Enrichment of biological pathway analysis of DEGs in a significant module.

Understanding interactions between DEGs that may be related to the development of SCLC is necessary to explore the underlying mechanisms in SCLC. PPI network was detected by STRING, as shown in Figure 4. A total of 224 nodes and 5302 edges, with each node representing a protein (gene) and each edge representing an interaction relationship were obtained. Genes with the most significant modules were screened by MCODE with score > 55 (Figure 4), and results indicated that the identified genes were all upregulated genes (Figure 5). The results of biological pathway analysis of significant module DEGs were shown in Figure 6 (Table S5); results showed that DEGs in significant module were mainly enriched in cell cycle, mitotic, and cell replication.

### 3.4. Hub Gene Selection and Analysis

**Figure 7:**
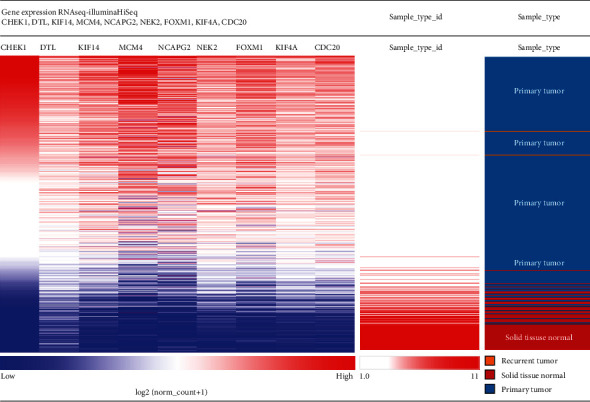
UCSC was utilized for constructing the hierarchical clustering of hub genes. The pink bands represent normal samples and the blue bands represent SCLC samples. The red markers indicate upregulated gene expression. Blue markers indicate downregulated genes.

Identification of 10 hub genes (score ≥ 69.5) was conducted, and the results are shown in Table 2. The hierarchical clustering results in Figure 7 reveal that the identified hub genes could distinguish SCLC samples from normal ones.

### 3.5. Prognostic Analysis of Hub Genes

**Figure 8:**
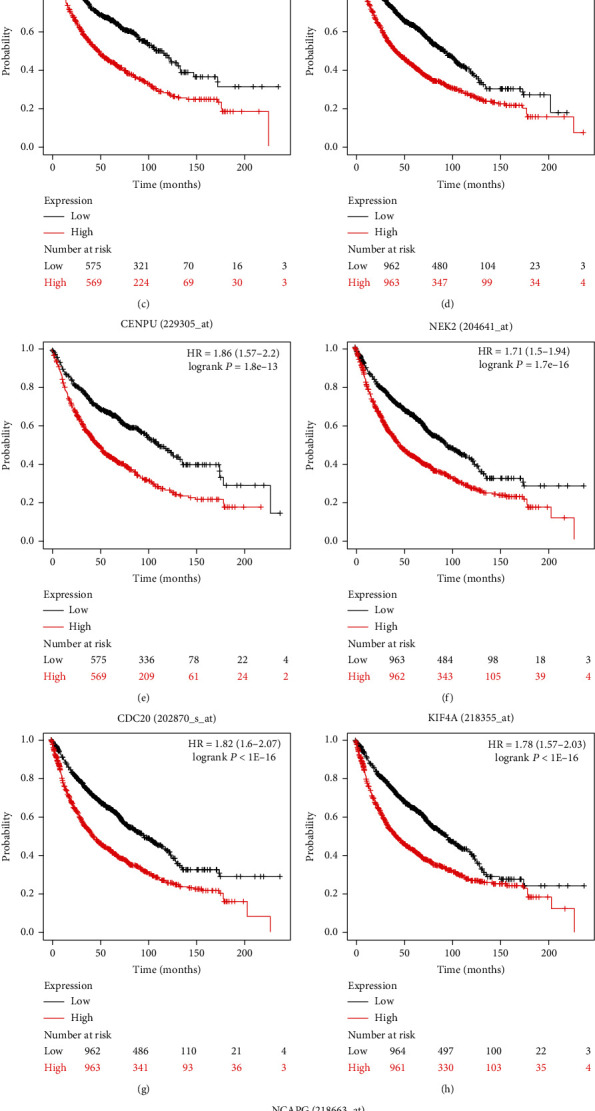
Kaplan-Meier curve analysis of the influence of hub genes on the prognosis of lung cancer patients. The statistical difference was considered significant if *P* < 0.05.

Kaplan-Meier analysis was conducted to analyze the relation between overall survival and the 10 hub genes identified earlier and predict the association of these genes with lung cancer prognosis. The median survival time of the group showing high expression of CHEK1, DTL, KIF14, MCM4, CENPU, NEK2 CDC20, KIF4A, and NCAPG2 was significantly shorter than that of the group demonstrating low expression of these genes, and the difference between groups was statistically significant (*P* < 0.05) as shown in Figure 8.

### 3.6. Hub Gene Analysis

**Figure 9:**
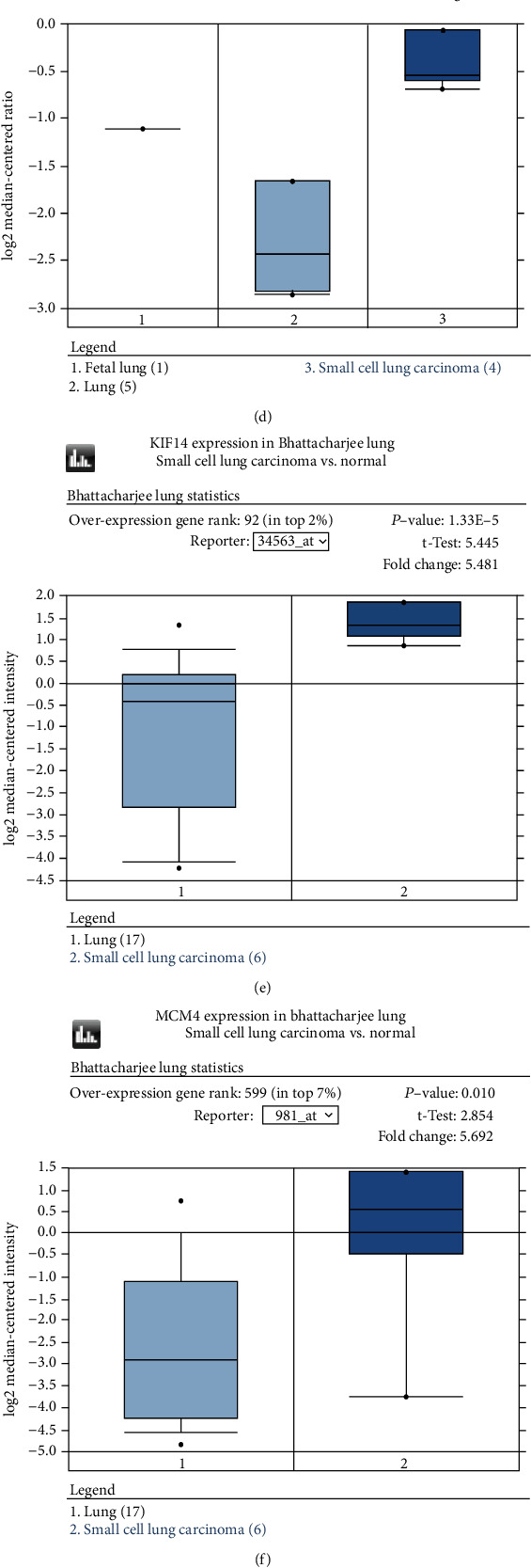
ONCOMINE analysis of hub gene expression in SCLC vs. adjacent tissue.

The ONCOMINE database could be utilized to determine the expression of hub genes in normal and SCLC tissues. The results in Figure 9 show that the hub genes (except KIF4A) were significantly overexpressed in the SCLC tissues of different studies.

## 4. Discussion

SCLC, a subtype of lung cancer, is the sixth most common cause of all cancer-related deaths worldwide and has significant clinical and pathological features, including early metastasis and poor prognosis. The survival time of patients in advanced stages of the disease is less than 1 year, and the 2-year survival rate is only approximately 5% [12]. The high drug resistance and recurrence rates of SCLC are mainly attributed to the high mutation rate of genes involved in this malignancy and genomic instability. Studies have shown that the mutation frequencies of P53 and RB1 in SCLC are 85% and 57%, respectively. Thus, mutations of P53 and RB1 indicate poor prognosis [13]. The molecular mechanisms of SCLC remain unclear, and identification of potential key genes that can serve as biomarkers is an urgent undertaking. Bioinformatics may be used to explore gene-level changes in SCLC and identify potential biomarkers.

In our study, three datasets, GSE30219, GSE99316, and GSE149507, were applied to screen for DEGs between SCLC and normal adjacent tissues, and a total of 268 DEGs were obtained. GO and KEGG enrichment analyses were performed on these 268 DEGs, and results indicated that the upregulated DEGs were mainly enriched in cell cycle, mitotic, DNA replication, mitotic M-M/G1 phases, M phase, mitotic prometaphase, and mitotic G1-G1/S phases and the downregulated DEGs were mainly enriched in epithelial-to-mesenchymal transition, serotonin degradation, noradrenaline and adrenaline degradation, FOXA transcription factor networks, and FOXA1 transcription factor network. Mutations in tumor cells often result in changes in the cell cycle leading to unrestricted growth compared with that of normal cells. Zhang et al. found that cell cycle inhibitors could be used in SCLC to interfere with the cell cycle, induce DNA replication stress and genomic instability, and trigger immune response signal [14, 15]. These studies showed consistency with the finding that dysregulation of the cell cycle promotes tumorigenesis and progression.

In the analysis of significant module genes, it was found that all 112 genes in cluster 1 were upregulated, and we selected the top 10 genes as hub genes. Genes with a score > 75 were selected as hub genes. CDC20, CENPU, CHEK1, DTL, KIF4A, KIF14, MCM4, NCAPG2, NEK2, and FOXM1 were all located in core nodes in the PPI network, which means these 10 genes may be critical therapeutic targets for SCLC.

CDC20, a homolog of Saccharomyces cerevisiae cellular division cycle 20 protein, serves as an activator of the anaphase-promoting complex, which performs an essential function in governing cell cycle progression for cell division [16]. CDC20 is highly overexpressed in NSCLC patients [17], and downregulation of CDC20 expression can slow down the growth and colony formation rate of lung cancer cells [18]. According to GO analysis results, CDC20 is mainly involved in the biological process of cell cycle; there is evidence that CDC20 regulates the cell cycle progression of cell division by targeting several key degradation substrates [18]. Therefore, our results are consistent with previous studies, suggesting that CDC20 may be a key factor in the onset and progression of SCLC.

Centromeric protein U (CENPU), also known as myelodysplastic/myeloid leukemia factor 1 Interaction protein (MLF1IP) [19], is an important component of spindle recovery after injury. CENPU is reported to be abnormally high expressed in various human tumor tissues and is involved in tumor progression, such as prostate cancer, breast cancer, bladder cancer, and ovarian cancer, and its overexpression has been shown to predict poor prognosis [20–22]. A study has demonstrated that CENPU regulated the proliferation and migration of lung adenocarcinoma cells through the PI3K/AKT pathway [23]; however, there are no data on the carcinogenic effect and clinical significance of CENPU in SCLC. Unfortunately, the biological process related to CENPU was not screened in our study (the result is unknown). In pathway analysis, CENPU was found to be involved in the PLK pathway in addition to the pathways related to cell cycle, DNA replication, and mitosis, which has not been verified by basic experiments.

CHEK1, an evolutionarily conserved serine/threonine kinase, has been shown to regulate cell cycle checkpoints, coordinating cellular activity involved in DNA repair, and cell cycle arrest [24]. A growing number of studies have found that CHEK1 is highly expressed in multiple cancer species [25–27] and is considered a potential target for cancer treatment. At present, there is little evidence on the relationship between CHEK1 and SCLC. Gali-Muhtasib et al. [27] found that CHEK1 was significantly overexpressed in SCLC compared with NSCLC samples and inhibited CHEK1 or ATR could induce genotoxic stress and apoptosis. Through our analysis, it was found that the biological process CHEK1 was mainly involved in was the cell cycle, and its related pathways included ATR and ATM signaling pathways in addition to the cell cycle [28, 29], of which is consistent with the reported CHEK signaling pathway. It is worth noting that CHEK1-mediated inactivation of Cyclin B is implicated in our analysis, which requires further verification.

Until now, DTL was thought to be involved in the regulation of cell cycle and DNA replication to influence tumor progression [30]. Notably, previous studies have shown that DTL expression is elevated in many cancers [31, 32], and the abnormal expression of DTL is also associated with poor prognosis [33]. However, systematic studies of the function of DTL in tumors remain to be evaluated.

KIF4A is one of the members of the kinesin superfamily. KIF4A is involved in a variety of cellular activities and plays a critical role in biological processes such as mitotic spindle formation and DNA damage repair [34]. Our analysis shows that KIF4A is involved in biological processes of regulation of nucleobase, nucleoside, nucleotide, and nucleic acid metabolism, which is consistent with the previous study [35]. A large number of studies have confirmed that KIF4A is overexpressed in colorectal cancer, liver cancer, and lung cancer [36–38] and is an independent prognostic risk factor [38]. It can also be seen from the analysis results of the GEO database that KIF4A is highly expressed in SCLC tissues, and its high expression is related to poor prognosis of patients, which proves that KIF4A can be used as a potential target for the treatment of SCLC.

Like KIF4A, KIF14 is a member of the driver superfamily. KIF14 is widely believed to play a role in tumorigenesis. The overexpression of KIF14 may lead to rapid and error-prone mitosis [39] and is involved in the progression of a variety of malignancies, such as retinoblastoma and gastric cancer [40, 41], providing evidence that KIF14 may be an oncogene in the progression of a variety of cancers, while in the study of lung adenocarcinoma, it was found that KIF14 was underexpressed in 30% of cancer tissue samples [42], and the decreased expression of KIF14 was significantly correlated with the overall survival rate of lung cancer patients [43]. However, it can be seen from our analysis that KIF14 is highly expressed in SCLC cancer tissues, reminding us that we can distinguish lung adenocarcinoma from SCLC by the level of KIF14 expression. KIF14 is mainly involved in cell growth and/or maintenance biological processes. However, it is a pity that the main signaling pathway of KIF14's major involvement in SCLC process has not been found, and further research needs to be carried out by future researchers.

MCM4 is a microchromosomal maintenance (MCM) protein complex, which is involved in cell cycle and cell replication. In cancer cells, abnormal expression of members of the MCM family has been reported in a wide range of cancers, and knockout of these genes can inhibit malignant phenotypes of cancer cells. Recent studies by Sanada et al. [44] have shown that siRNA-mediated MCM4 knockdown attenuates the invasiveness of lung adenocarcinoma cells. In addition, their team also confirmed that siRNA-mediated MCM4 knockdown enhanced the sensitivity of SCLC cells to cisplatin [45], suggesting that MCM4 could be used as a therapeutic target for SCLC.

The non-SMC lectin II complex subunit G2 (NCAPG2), a component of the lectin II complex, interacts with PLK1 to regulate correct chromosome separation. Similar to our results, NCAPG2 is highly expressed in a variety of cancers, such as liver cancer and NSCLC [46, 47]. According to our analysis, its overexpression leads to a short survival time, which is consistent with the results of previous studies in other cancer type [47]. At the same time, we also confirmed that NACPG2 affects tumor process by participating in cell growth, and Meng et al. also proposed the conclusion that NCAPG2 can affect cell proliferation in their study [46]. These findings confirm that NCAPG2 is both an oncogene of SCLC and a biomarker that predicts poor prognosis in patients.

Never in mitosis- (NIMA-) related kinase 2 (NEK2) is a member of the serine/threonine kinase family, mainly involved in regulating the cell cycle progression and microtubule organization and stabilization [47]. Recent reports have consistently identified high expression of NEK2 in various cancer types, including breast cancer, cervical cancer, liver cancer, and lung cancer [48–52]. In addition, NEK2 overexpression was enhanced in advanced lung adenocarcinoma, suggesting a role for NEK2 in tumor progression [53]. In terms of overall survival, patients with high NEK2 expression in NSCLC and its subtypes of lung adenocarcinoma have a poor prognosis [54, 55]. However, there are few related studies on NEK in SCLC, and further clinical studies and basic researches are needed. Therefore, the expression of NEK2 may be helpful in determining tumor progression and disease prognosis.

Forkhead Box M1 (FOXM1) is a member of the Forkhead family of proteins and is involved in cell cycle regulation. FOXM1 expression was low in quiescent cells but elevated in most tumors, including liver cancer and gastric cancer [56, 57]. Moreover, Hu et al. [58] found that FOXM1 and KIF4A proteins were upregulated in clinical liver cancer tissue samples, which was positively correlated with poor prognosis of patients with hepatocellular carcinoma. Currently, there are few studies on the biological function and clinical significance of FOXM1 in SCLC. Notably, Liang et al. [59] found that FOXM1 knockout inhibited SCLC formation in mouse models through increased levels of neuroendocrine markers Ascl1 and Cgrp and decreased levels of Yap1. In addition, this study also confirmed that SCLC with high FOXM1 expression was significantly associated with reduced clinical stage, extracthoracic metastasis, and OS with shorter progression-free survival. These evidences support the application of FOXM1 as a prognostic biomarker and potential molecular target for SCLC.

According to the Oncomine analysis results, the 10 hub genes identified in this work were able to distinguish SCLC samples from normal ones, thus suggesting their potential use as diagnostic biomarkers. We used Kaplan-Meier analysis to obtain the relations between these hub genes and lung cancer prognosis and found that the median survival time of the group with high expression of CDC20, CENPU, CHEK1, DTL, KIF14, MCM4, NCAPG2, NEK2, and FOXM1 is significantly shorter than that of the group with low expression of these genes. This finding suggests that the overexpression of these genes could predict the poor prognosis of patients with SCLC.

At present, several scholars have studied the GEO database of SCLC and obtained a certain number of DEGs. Liao et al. [60] screened out 5 highly expressed hub genes (NDC80, BUB1B, PLK1, CDC20, and MAD2L1) from 4 datasets (GSE60052, GSE43346, GSE15240, and GSE6044), and the cell cycle pathway was considered to be the main pathway for the diagnosis and treatment of SCLC of these five hub genes. Mao et al. [9] studied two databases, namely, GSE6044 and GSE19945, and finally identified 32 miRNAs and 32 regulated genes by using the bioinformatics platform “miRNAWalk.” It was suggested that bioinformatics analysis may contribute to a better understanding of the roles of DEGs, DEM, and miRNA genes in cell proliferation and signal transduction, and their related hub genes can be used as biomarkers for diagnosis and prognosis of SCLC, as well as potential drug targets. Wen et al. [61] studied two databases (GSE11969 and GSE6044) and finally confirmed 10 hub genes (TOP2A, PCNA, RFC4, CHEK1, TYMS, MCM2, CDC20, CDKN3, MCM3, and CDC6). At the same time, the signaling pathways of these 10 hub genes were also analyzed to provide molecular targets and diagnostic markers for the treatment and early diagnosis of SCLC. In the latest study, Chen et al. [11] studied 3 databases, namely, GSE40275, GSE99316, and GSE6052, and used GEO2R analysis tool to filter DEGs and Funrich for functional annotation; then, 8 hub genes (CDC20, BUB1, TOP2A, RRM2, CCNA2, UBE2C, MAD2L1, and BUB1B) were identified by the PPI network, module analysis, and mRNA expression level verification of hub genes in the ONCOMINE database. RT-qPCR was also used in clinical samples to verify that these hub genes may become prognostic markers or therapeutic targets for SCLC.

At present, the sample size of SCLC mRNA tissue in GEO database is relatively limited. GSE6044 and GSE60052 have been studied for several times. We need to analyze more datasets in the database (with a large enough sample size) to expand the number of DEGs. Therefore, new datasets GSE30219, GSE9316, and GSE149507 were selected for analysis in this study. Our innovation lies in the prognostic analysis, namely, the survival curve analysis, of the newly screened 10 hub genes, which improves the clinical application value of hub genes. But there are limitations to our study. Firstly, the data used in this study were all from public databases, but the quality of the data could not be evaluated. Secondly, the sample size of relevant data is relatively small. Third, it has not been validated in clinical samples. Therefore, a lot of valuable biological information may be ignored in our research. Finally, all 10 hub genes are overexpressed in SCLC, but the related mechanisms have not been fully clarified. Therefore, more molecular evidence is needed. In addition, current SCLC studies lack prognostic data of these hub genes, such as survival curves, which limits the clinical application value of hub genes. In this paper, the expression levels of 8 pivotal genes were analyzed. Whether these hub genes can be used as biomarkers or therapeutic targets for SCLC needs further study.

## 5. Conclusion

In summary, we aimed to find DEGs associated with the carcinogenesis and progression of SCLC. The DEGs we obtained revealed a significant function in the occurrence and metastasis of SCLC. This work provides new molecular targets at the genetic level, as well as new insights into precision SCLC treatment. Further experiments are necessary to verify the results.

## Figures and Tables

**Table 1:** 268 DEG lists were identified and confirmed from three GEO datasets.

Regulation	Number	Genes
Upregulation	192	TPX2, GF2BP3, CCNB1, PCSK1, CXCL13, GINS1, ZIC2, ISL1, ANLN, BIRC5, KCNC1, KNTC1, CCNE1, FOXM1, CDK1, RACGAP1, TOX3, CDC6, CENPU, CEL, AURKA, KIF14, MAD2L1, ZNF711, RNF182, IGSF9, ELAVL2, KIF4A, MCM10, TYMS, DNA2, MELK, ZWINT, NDC80, PPM1E, OIP5, CCNA2, GTSE1, POU3F2, NUF2, PTTG1, NRTN, MMP12, CDCA5, UBE2T, CKS2, FOXG1, ECT2, DEPDC1, KIF23, NOL4, CENPE, SPAG5, TUBB2B, INSM1, HOXD10, CCNB2, PRC1, SRD5A1, CDT1, CENPW, CEP55, PLEKHG4B, CCNE2, CDC45, PMAIP1, MCM2, MCM4, INHBE, SBK1, DLGAP5, RAD51AP1, SCG3, KIF1A, CKAP2L, MKI67, CDCA2, CDCA8, EXO1, GRP, C12orf56, FZD3, NMU, RGS17, CENPK, LOC646903, UGT8, NEK2, SOX2, GINS2, ESPL1, DONSON, AMER2, E2F8, MEX3B, DEPDC1B, CLGN, DSP, SOX4, ADAMDEC1, UCHL1, EZH2, NUP62CL, CHEK1, NEIL3, KIF11, KIF18B, ASF1B, RIPPLY3, CDH2, KIFC1, FOXO6, DLX6, NCAPG2, KIF2C, GNG4, FBXO5, C5orf34, HAGLROS, CDCA7, CDC20, RFC4, MIAT, BUB1, PBK, AP3B2, SKA1, TRIP13, CDC7, NCAPH, ACYP1, SMC2, RMI1, ASPM, CDCA3, ATAD2, BRIP1, ELAVL4, STIL, UBE2C, MND1, NELL1, RAB3B, HES6, POLE2, RRM2, TOP2A, FEN1, HELLS, FANCI, RAD54L, DLX5, ZNF367, SPC25, KIF18A, DDC, KIF15, BUB1B, HJURP, DTL, FAM83D, CENPV, MEST, HMMR, NRCAM, RIMS2, GMNN, KIF20A, ORC6, KCNMB2, SOX11, SIX1, HEPACAM2, ASCL1, PEX5L, SGO2, CDKN2A, IGFBPL1, LHX2, ONECUT2, TTK, CDKN3, GAD1, NCAPG, RASSF6, CELF4, RMI2, RNF183, CENPF, NUSAP1, ELAVL3, ST18
Downregulation	76	FIBIN, PPP1R14A, GHR, MAOA, GDF10, LAMP3, FCN1, HLF, FHL1, RRAD, SCN4B, SFTPD, PLAC9, FHL5, GPM6A, NR3C2, EMCN, GPIHBP1, OGN, SCGB1A1, CA4, AQP3, LYVE1, ADAMTS8, NAPSA, ADIRF, SRPX, RNASE1, SFTPC, FXYD1, FOSB, RSPO3, CX3CR1, WFDC1, BMP5, AOX1, CFD, RNASE4, CHRDL1, DPP4, MUC1, CD36, SERPINA1, TNNC1, ABCA8, STEAP4, AOC3, FCER1A, ZBTB16, ASPA, FGFR2, EDNRB, C7, DPT, MFAP4, SCARA5, MAOB, PTGDS, CDO1, ADAMTS1, S1PR1, NEXN, CA2, SFTPB, ANPEP, AQP1, RBMS3, ADH1B, NEDD9, CXCL5, FABP4, FAM107A, GPX3, SCGB3A2, GPAT3, FBLN5

**Table 2:** Top 10 hub DEGs with high score.

Gene symbol	Score	Type	MCODE cluster
CDC20	69.5	Upregulated	Cluster 1
CENPU	69.7	Upregulated	Cluster 1
CHEK1	70.2	Upregulated	Cluster 1
DTL	70.2	Upregulated	Cluster 1
KIF4A	69.5	Upregulated	Cluster 1
KIF14	70.1	Upregulated	Cluster 1
MCM4	69.9	Upregulated	Cluster 1
NCAPG2	69.7	Upregulated	Cluster 1
NEK2	69.6	Upregulated	Cluster 1
FOXM1	69.5	Upregulated	Cluster 1

## Data Availability

The datasets analyzed during current study are available from the corresponding author upon reasonable request.

## Supplementary Materials

Supplementary Materials Table S1: gene expression microarray datasets for SCLC in GEO datasets, including GSE30219, GSE99316, and GSE149507. Table S2: information for the DEGs identified from the GEO datasets (log_2_FC ≥ 1, *P* < 0.05). Table S3: enrichment analysis of the upregulated DEGs. Table S4: enrichment analysis of the downregulated DEGs. Table S5: enrichment analysis of the DEGs of significant module.
